# Association of Renal Function and Menopausal Status with Bone Mineral Density in Middle-aged Women

**DOI:** 10.1038/srep14956

**Published:** 2015-10-13

**Authors:** Yueh-Hsuan Sheng, Jen-Hau Chen, Jeng-Min Chiou, Keh-Sung Tsai, Yue-Yuan Lee, Chwen-Keng Tsao, Yen-Ching Chen

**Affiliations:** 1 Department of Family Medicine, Renai Branch, Taipei City Hospital, Taipei, Taiwan; 2 Institute of Epidemiology and Preventive Medicine, College of Public Health, National Taiwan University, Taipei, Taiwan; 3 Department of Geriatrics and Gerontology, National Taiwan University Hospital, Taipei, Taiwan; 4 Institute of Statistical Science Academia Sinica, Taipei, Taiwan; 5 Department of Internal Medicine and Laboratory Medicine, National Taiwan University Hospital, Taipei, Taiwan; 6 MJ Health Management Institution, Taipei, Taiwan; 7 Department of Public Health, Taipei, Taiwan; 8 Research Center for Genes, Environment and Human Health, College of Public Health, National Taiwan University, Taipei, Taiwan

## Abstract

The association between mild renal dysfunction and bone mineral density (BMD) has not been fully explored. It is also unclear how menopausal status and the use of Chinese herb affect this association. This is a cross-sectional study that included a total of 1,419 women aged 40 to 55 years old who were recruited from the MJ Health Management Institution in Taiwan between 2009 and 2010. Spinal BMD was assessed by dual-energy X-ray absorptiometry. Renal function was assessed using estimated glomerular filtration rate (eGFR) and creatinine clearance rate (CCr). The multivariable logistic regression and general linear models were employed to assess the association between renal function and BMD. Stratification analyses were performed by menopausal status and use of Chinese herbs. Low CCr levels were significantly associated with low BMD [adjusted odds ratio (AOR) = 1.48, 95% confidence interval (CI) = 1.15–1.90]. This association was observed in premenopausal women (AOR = 1.43, 95% CI = 1.07–1.92) and in women not taking Chinese herbs (AOR = 1.48, 95% CI = 1.14–1.94). CCr is a better predictor for low BMD in middle-aged women. Menopausal status and the use of Chinese herbs also affected this association.

Osteoporosis is a disease characterized by low bone mass and loss of bone tissue, resulting in an increased risk of fractures in the spine, hip, and wrist^1^. According to the US National Health and Nutrition Examination Survey (2005–2008), the prevalence of spinal osteoporosis in the elderly was highest among Mexican Americans (women: 24.4%, men: 4.6%), followed by Caucasian Americans (women: 10.9%, men: 2.2%), and African Americans (women: 5.3%, men: data not shown)^2^. The Nutrition and Health Survey of Taiwan (2005–2008) reported that spinal osteoporosis occurs in 12.6% and 4.3% of women and men of the same age group, respectively^3^. Osteoporosis is currently considered as an important disease among the elderly.

About 61% and 24% of Caucasian women with osteoporosis have mild to moderate and severe renal disease, respectively^4^. Patients with chronic kidney disease (CKD) lose the capacity to excrete adequate phosphates and fail to convert vitamin D to 1,25-dihydroxyvitamin D^5^. These conditions contribute to the development of secondary hyperparathyroidism and the elevation of fibroblast growth factor (FGF)-23^6^, resulting in the release of calcium from the bones and a decrease in bone formation. These characteristics are considered symptoms of a CKD-mineral bone disorder.

Previous studies have shown that dialysis patients are at an increased risk for osteoporosis and hip fractures^7^,^8^. However, studies exploring “mild” renal dysfunction (e.g., CKD stages 1 and 2 or decreased renal function) are limited. Earlier reports have reported that decreased renal function was associated with low bone mineral density (BMD) or osteoporosis^9^,^10^,^11^,^12^; however, these studies were mostly based on small study populations (N = 27 to 659). The use of different methods to estimate renal function [e.g., Cockcroft-Gault (C-G) equation for creatinine clearance rate (CCr) or modification of diet in renal disease (MDRD) equation for estimated glomerular filtration rate (eGFR)] has also led to inconsistencies in the results. In addition, past studies included postmenopausal women only^4^,^9^,^10^,^11^,^12^, which does not allow the assessment of the effects of menopause on BMD.

In sum, past studies have focused on severe renal dysfunction and postmenopausal women. To screen low BMD risk at an early age, this study aimed to explore the association of mild renal dysfunction with BMD in middle-aged women. Chinese herbs are known to play an important role in CKD in the Chinese population^13^,^14^ because of some harmful ingredients in them. In addition, menopause is a major predictor of BMD. Therefore, this study further assessed how menopausal status and the use of Chinese herbs affected the association between renal function and low BMD.

## Materials and Methods

### Study population

A cross-sectional study was conducted, which consisted of a total of 1,575 Taiwanese women aged 40 to 55 years and underwent health examination at the MJ Health Management Institution in Taipei, Taiwan between 2009 and 2010. Participants with the following conditions or diseases were excluded (N = 156): (1) diseases known to affect BMD levels, e.g., hyperparathyroidism, hyperthyroidism, type 1 diabetes, inflammatory bowel disease, chronic active hepatitis, liver cirrhosis, chronic cholestatic diseases, and multiple myeloma (n = 2), (2) medications known to affect BMD, e.g., hormone replacement therapy (n = 60), steroids (n = 6), (3) low BMD of the lumbar spine (n = 86), and (4) lack of blood samples or data for the calculation of eGFR or CCr (n = 2). After exclusion, a total of 1,419 women were included for data analyses. Informed consent was obtained from each participant. The institutional review boards of the MJ Health Management Institution and National Taiwan University approved the study protocol. The present study complied with the guidelines of the World Medical Association Declaration of Helsinki.

A self-reported questionnaire was used to collect information on lifestyle [e.g., smoking, alcohol consumption, and regular exercise (≥30 min for 2 to 3 days per week)], menopausal status, disease history (e.g., hypertension and diabetes), and medication history. Menopause was defined as a woman who did not have a period for 12 months, which is not attributable to other biological or physiological causes^15^.

### Determination of bone mineral density

BMD (g/cm^2^) of the lumbar spine was measured using dual-energy X-ray absorptiometry (DXA, GE Lunar Health Care, DPX-L, USA), which was calibrated using a standard automated test program provided by the manufacturer.

### Assessment of renal function and biochemical measurements

eGFR and CCr were used to estimate renal function. According to the 2003 Guidelines of the National Kidney Foundation-Kidney Disease Outcomes Quality Initiative (NKF-KDOQI), the MDRD equation and C-G equation were recommended for the assessment of CKD. A simplified MDRD equation was used to estimate GFR (mL/min/1.73 m^2^) = 186 × [serum creatinine] − 1.154 × [age] − 0.203 (×0.742 if female). The C-G equation was used to estimate CCr (mL/min) = [(140 − Age) × weight (kg)] (×0.85 if female)/[(serum creatinine) × 72].

Obesity has been known to affect BMD and osteoporosis^16^. Therefore, measurements relating to the lipid profile of the study participants were collected, e.g., total cholesterol, low-density lipoprotein cholesterol (LDL-C), high-density lipoprotein cholesterol, triglyceride, alkaline phosphatase (ALP), fasting glucose (FG), serum creatinine, and uric acid (UA).

### Statistical analyses

**Figure 1:**
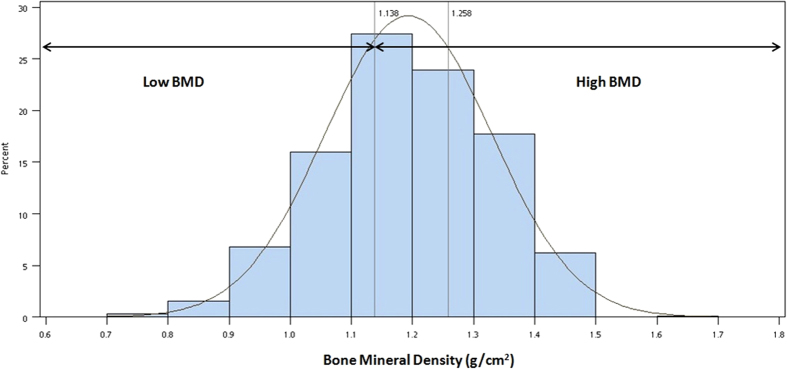
Distribution and tertiles of BMD. T1: BMD < 1.138 g/cm^2^; T2: 1.138 g/cm^2^ ≤ BMD < 1.258 g/cm^2^; T3: BMD ≥ 1.258 g/cm^2^. High BMD indicates T2+T3; low BMD indicates T1. Abbreviations: BMD, bone mineral density; T, tertile.

The Student’s t test (for normally-distributed continuous variables), Mann-Whitney U test (for non-normally distributed continuous variables), and χ^2^ test (for categorical variables) were used to compare the distribution of potential confounders by high and low BMD (T2+T3 vs. T1, defined as below). In addition to the use of continuous BMD variables for data analysis, BMD was further tertiled (T1, T2, and T3) to identify a susceptible population in terms of public health. High BMD was defined as T2 plus T3, and low BMD was defined as T1 (Fig. 1). Grouping was conducted as described in our recent publication^17^ and other studies^18^,^19^. In the present study, only a small portion of participants had abnormal eGFR value when clinical cutoff point was applied (eGFR < 60 mL/min/1.73 m^2^: 1.1%) because middle-age women are relatively healthy than old women. In addition, our goal is to assess if preclinical renal dysfunction is related to low BMD. Therefore, clinical cutoffs may not be suitable for this population. Similar approaches using Youden’s index to identify cutoff points for a study population were commonly used previously^17^,^20^. A general linear regression model was used to estimate adjusted BMD (g/cm^2^) by renal function. A logistic regression model was used to estimate adjusted odds ratios (AORs) and 95% confidence intervals (CIs) in women with low versus high BMD for eGFR and CCr values, respectively. An ordinal logistic regression model was also applied when treating BMD as an ordinal variable (T1, T2, and T3).

Factors known to affect BMD or osteoporosis were adjusted in the model, e.g., age, menopausal status, body weight, serum ALP, LDL-C, FG, hypertension, use of Chinese herb, UA, and body height. Because few participants had abnormal serum ALP values (20 to 140 IU: 0.14%) and no clinical cutoff values were available for body weight and height, these variables were treated as continuous variables in the model.

The impact of menopausal status (yes/no) was explored by generating a product term of renal function and important covariates (e.g., menopausal status and the use of Chinese herb) in the regression models. Stratified analysis was performed based on menopausal status and the use of Chinese herbs, respectively, to establish the relationship between eGFR or CCr and BMD. All analyses were performed using SAS v 9.3 (SAS Institute, Cary, NC). All statistical tests were two-sided and *P* values < 0.05 were considered statistically significant.

## Results

### Characteristics of the study population

The present study included a total of 1,419 women. Compared to women with high BMD values, women with low BMD levels were older (47.8 vs. 45.7 years old), had a lower body weight (53.5 vs. 57.1 kg), higher LDL-C values (111.6 vs. 107.1 mg/dL), lower FG values (96.9 vs. 99.1 mg/dL), higher ALP levels (65.1 vs. 56.8 IU), lower CCr values (74.8 vs. 80.5 mL/min), and more participants had experienced menopause (39% vs. 13%, Table 1). The distribution of UA, eGFR, use of Chinese herbs, history of hypertension, cigarette smoking, alcohol consumption, calcium supplement intake, and regular exercise were similar between the high and low BMD groups. eGFR was negatively correlated with BMD, body weight, and UA (age-adjusted Spearman correlation coefficient: −0.25 to −0.06, *P* < 0.05). In contrast, CCr was positively correlated with BMD, body weight, hypertension, LDL-C, FG, ALP, UA, and eGFR (age-adjusted Spearman correlation coefficient: 0.07 to 0.72, *P* < 0.05).

### Association between renal function (eGFR or CCr) and BMD

Low eGFR values protected against low BMD [<80 vs. ≥80 mL/min/1.73 m^2^: AOR = 0.69, 95% CI = 0.53–0.89, Table 2]. Low CCr values were associated with an increased risk for low BMD (<78 vs. ≥78 mL/min, AOR = 1.48, 95% CI = 1.15–1.90, Table 2). The logistic regression models (binary BMD) and the ordinal logistic regression models (tertiled BMD) showed similar results (eGFR: <80 vs. ≥80 mL/min/1.73 m^2^: AOR = 0.64, 95% CI = 0.52–0.80; CCr: <78 vs. ≥78 mL/min, AOR = 1.42, 95% CI = 1.15–1.75).

### Adjusted mean BMD according to high and low renal function

After adjusting for other covariates, the mean BMD level was significantly lower in women with high eGFR values (high vs. low eGFR: 1.18 vs. 1.21 g/cm^2^, *P* = 0.001, Table 3); the opposite trend was observed for CCr (high vs. low CCr: 1.22 vs. 1.17 g/cm^2^, *P* < 0.0001, Table 3).

### Stratification by menopausal status

No significant interaction was observed between renal function (eGFR or CCr) and menopausal status (*P*_interaction_ = 0.21 and 0.60, respectively, Table 2). After stratification by menopausal status, low eGFR values showed a protective effect against low BMD in premenopausal women (<80 vs. ≥80 mL/min/1.73 m^2^: AOR = 0.63, 95% CI = 0.46–0.86, Table 2). In contrast, low CCr values showed an increased risk of low BMD in premenopausal women (<78 vs. ≥78 mL/min: AOR = 1.43, 95% CI = 1.07–1.92, Table 2).

After adjusting for other covariates, among premenopausal women, the mean BMD level was significantly lower in those with high eGFR levels (1.20 g/cm^2^) compared with those with low eGFR (1.24 g/cm^2^, *P* < 0.0001, Table 3); no significant difference in BMD levels was observed in postmenopausal women. In terms of CCr, BMD levels were significantly lower in premenopausal with low CCr (1.20 g/cm^2^) compared with premenopausal women with high CCr (1.23 g/cm^2^, *P* < 0.0001). Similar findings were found in postmenopausal women (high CCr: BMD = 1.14 g/cm^2^, low CCr: BMD = 1.09 g /cm^2^, *P* = 0.001).

### Stratification by the use of Chinese herbs

The use of Chinese herbs significantly influenced the association between eGFR and the risk of low BMD (*P*_interaction_ = 0.03). Among women not using Chinese herbs, low eGFR protected against low BMD (AOR = 0.62, 95% CI = 0.47–0.82, Table 4) compared with those with high eGFR. In contrast, in women not using Chinese herbs, low CCr was associated with an increased risk of low BMD (AOR = 1.48, 95% CI = 1.14–1.94, Table 4).

After adjusting for other covariates, among women not using Chinese herbs, the mean BMD level was significantly lower in those with high eGFR (1.19 g/cm^2^) compared with those with low eGFR (1.21 g/cm^2^, *P* = 0.001, Table 5). In contrast, among women not using Chinese herbs, BMD levels were significantly lower in those with low CCr (1.17 g/cm^2^) compared with those with high CCr (1.22 g/cm^2^, *P* < 0.0001, Table 5).

## Discussion

Previous studies have shown that advanced CKD was associated with low BMD levels and accelerated bone loss^21^,^22^. However, no study has assessed the effectiveness of using mild renal dysfunction in predicting low BMD at an early age. Therefore, this study was aimed to determine this association in a middle-aged, healthy female population. The present study observed that low CCr values were associated with an increased risk of low BMD in premenopausal women (AOR = 1.43); however, previous Caucasian and Japanese studies observed this association in postmenopausal women^9^,^11^. In contrast, low eGFR protected against low BMD in premenopausal women (AOR = 0.63), which has not been explored previously. Decreased renal function has been associated with vitamin D deficiency, hyperphosphatemia, and hyperparathyroidism^5^, which in turn results in the release of calcium from bones and a decrease in the rate bone formation. The inconsistencies between the present study and previous investigations might be attributable to the severity of impaired renal function (healthy people *vs.* advanced CKD patients) and the study population (pre- and postmenopausal women in this study *vs.* postmenopausal women only in previous studies). Although the C-G and MDRD equations generated similar predictions on renal function among Asians^23^,^24^, the contradictory findings between eGFR and CCr in predicting low BMD risk might be attributable to the inclusion of weight, which is an important BMD predictor, in the C-G equation. Both body weight and body mass index (BMI) had major effects on BMD especially weight-bearing bones in women^25^. In our study, overweight showed a protective effect on low BMD (BMI ≥ 24 vs. 18.5 ≤ BMI < 24, AOR = 0.42, 95% CI = 0.30–0.59). While low BMI was associated with higher risk of low BMD (BMI < 18.5 vs. 18.5 ≤ BMI < 24, AOR = 2.69, 95% CI = 1.61–4.49). Because either BMI or weight is collinear with CCr, statistical models assessing the association between CCr and BMD did not adjust for these variables. Therefore, the findings of the present study indicate that CCr is a better predictor of low BMD compared to eGFR. The following discussion focuses on the findings relating to CCr.

Estrogen induces osteoclast apoptosis, inhibits osteoblast apoptosis, and blocks the formation of new osteoclasts to prevent bone loss^26^,^27^. Menopausal status is considered an important factor in the maintenance of BMD levels, especially when estrogen levels decrease during menopause. To the best of our knowledge, no study has evaluated the effect of menopausal status or the use of Chinese herbs on the association between renal function and BMD. This study has shown that low CCr values are associated with an increased risk of low BMD in premenopausal women, and this effect was further enhanced in postmenopausal women. It is possible that the reduction in estradiol levels might have led to renal injury via its effects on the renin-angiotensin system, wherein rennin levels are elevated in postmenopausal women^28^. Furthermore, the stronger association observed in postmenopausal women might be a result of accelerated bone loss during late perimenopause and postmenopausal years, whereas minimal changes occur during pre- and early perimenopause^29^. Additional studies clarifying the interaction between menopause and renal function on BMD are therefore warranted.

Chinese herb is a mixture of different medicinal plants or animal parts. Reports have shown that some Chinese herbs significantly affect renal function^30^. Ingredients like ephedra used in slimming pills is composed in Chinese herb and can cause nephrolithiasis, while heavy metals (cadmium, arsenic, lead) in contaminated soil where herbs grow up can induce renal dysfunction^30^,^31^. Another composition aristolochic acid is a strong carcinogen that can cause kidney interstitial fibrosis, impaired kidney function, urothelial atypias, tumors of the urinary tract, and Chinese herb nephropathy^14^,^32^,^33^,^34^. A Taiwanese study observed that the regular use of Chinese herbs markedly increased the risk of CKD and all-cause mortality^35^. These properties could explain our findings that low CCr levels were associated with a low BMD risk in women not using Chinese herbs. This association slightly increased but did not reach statistical significance among women who used Chinese herbs, probably because of the small sample size in this subgroup (N = 120). The present study observed that, in women not using Chinese herbs, BMD was significantly higher in those with higher CCr (1.22 g/cm^2^) than those with low CCr (1.17 g/cm^2^, *P* < 0.0001). However, this difference did not reach statistical significance among women using Chinese herbs (high CCr: BMD = 1.18 g/cm^2^, low CCr: BMD = 1.15 g/cm^2^, *P* = 0.28). In addition, BMD levels were lower in women using Chinese herbs (1.16 g/cm^2^) compared to those who did not (1.20 g/cm^2^, *P* = 0.005). It is possible that women with low BMD take Chinese herbs to improve their health. However, low BMD might also be the effect of using Chinese herbs. Because Chinese herbs contain various ingredients that have different effects on BMD, assessing the effects of Chinese herbs on renal function and BMD might be more complicated than previously assumed.

The present study has several strengths. First, the sample size (N = 1,419) is relatively large compared to most previous studies (N = 27 to 659). Second, this study included both pre- and postmenopausal women and thus allowed us to explore how menopausal status influenced the association between renal function and BMD. Third, Chinese herbs are considered as the leading cause of renal insufficiency in Taiwanese people because of some harmful ingredients in them. The inclusion of Chinese herbs in the analysis has helped us clarify the association between renal function and BMD in this Chinese population. The present study also has some limitations. First, this study used a cross-sectional design and thus the causal inference between renal function and BMD was unavailable. In addition, information on the use of Chinese herbs was self-reported, which no further details on the types of herbs and their corresponding doses utilized.

In summary, the present study has shown that CCr is a better predictor of BMD than eGFR because the former incorporated the weight variable, which is an important predictor of BMD, into the equation. Low CCr values were associated with a further increase in the risk of low BMD in postmenopausal women and in women using Chinese herbs. Chinese herbs are commonly used in this population and have been related to poor renal function. It is thus possible that some medications (e.g., bisphosphonates) for the treatment of low BMD might not be appropriate for patients with low renal function (CCr < 35 mL/min)^36^ . Therefore, physicians should consider renal function during the design of a treatment regimen for female patients with low BMD. Prospective studies exploring the role of CCr in predicting low BMD risk and in designing the appropriate treatment of patients are therefore warranted.

## Additional Information

**How to cite this article**: Sheng, Y.-H. *et al.* Association of Renal Function and Menopausal Status with Bone Mineral Density in Middle-aged Women. *Sci. Rep.* **5**, 14956; doi: 10.1038/srep14956 (2015).

## Figures and Tables

**Table 1:** Characteristics of the study population.

Variables	High BMD (≥1.138 g/cm^2^) (n = 951)	Low BMD (<1.138 g/cm^2^) (n = 468)	*P*^a^
Mean ± S.D.			
Age (years)	45.7 ± 4.0	47.8 ± 4.6	<0.0001
Body weight (kg)	57.1 ± 8.7	53.5 ± 7.7	<0.0001
LDL-C (mg/dL)	107.1 ± 27.9	111.6 ± 28.4	0.005
Fasting glucose (mg/dL)	99.1 ± 18.0	96.9 ± 13.8	0.01
Alkaline phosphatase (IU)	56.8 ± 15.8	65.1 ± 19.2	<0.0001
Uric acid (mg/dL)	4.6 ± 1.0	4.6 ± 1.0	0.67
eGFR (mL/min/1.73 m^2^)	83.6 ± 11.6	84.5 ± 12.0	0.23
CCr (mL/min)	80.5 ± 15.5	74.8 ± 13.7	<0.0001
n (%)			
Postmenopausal status	122 (13)	180 (39)	<0.0001
Use Chinese herb	72 (8)	48 (10)	0.09
Hypertension	138 (15)	56 (12)	0.19
Ever smoker	79 (8)	36 (8)	0.79
Alcohol consumption	57 (6)	29 (6)	0.78
Calcium supplement	489 (51)	256 (55)	0.27
Regular exercise^b^	419 (44)	198 (42)	0.89

Abbreviations: LDL-C, low-density lipoprotein cholesterol; S.D., standard deviation; BMD, bone mineral density; eGFR, estimated glomerular filtration rate; CCr, creatinine clearance rate.

^a^*P*-values were obtained from Student’s t tests (normally-distributed continuous variables) and Chi-square tests (categorical variables) for comparing participants with high to low BMD.

^b^Regular exercise was defined as taking exercise ≥30 min for 2 to 3 days per week.

**Table 2:** Association between renal function (eGFR or CCr) and bone mineral density (low vs. high) by menopausal status.

Renal function	Overall (n = 1,419)		Premenopause (n = 1,101)		Postmenopause (n = 302)		*P*_*interaction*_
	n (L/H)	AOR (95% CI)	n (L/H)	AOR (95% CI)	n (L/H)	AOR (95% CI)	
eGFR (mL/min/1.73 m^2^)							
≥80^a^	306/579	1.00	209/511	1.00	94/62	1.00	0.21
<80^a^	162/372	**0.69 (0.53**–**0.89)**	75/306	**0.63 (0.46**–**0.86)**	86/60	0.85 (0.51–1.44)	
CCr (mL/min)							
≥78^a^	171/490	1.00	117/428	1.00	51/54	1.00	0.60
<78^a^	297/461	**1.48 (1.15**–**1.90)**	167/389	**1.43 (1.07**–**1.92)**	129/68	1.53 (0.89–2.63)	

Abbreviations: L, low BMD; H, high BMD; AOR, adjusted odds ratio; CI, confidence interval; BMD, bone mineral density; eGFR, estimated glomerular filtration rate; CCr, creatinine clearance rate.

All models were adjusted for age, menopausal status (yes/no), body weight (for eGFR model only), hypertension, low-density lipoprotein cholesterol, fasting glucose, serum alkaline phosphatase, serum uric acid, height (for CCr model only), and use of Chinese herb (yes/no).

Numbers in bold indicated statistical significant findings.

^a^The cutoff values of eGFR (80 mL/min/1.73 m^2^) and CCr (78 mL/min) were determined by maximizing the Youden’s index.

**Table 3:** Adjusted mean BMD level by high and low renal function (eGFR or CCr) and menopausal status.

Renal function	Overall (n = 1,419)			Premenopause (n = 1,101)			Postmenopause (n = 302)			*P*_*interaction*_
	n	Adjusted BMD^a^ Mean ± S.E. (g/cm^2^)	*P*^c^	n	Adjusted BMD Mean ± S.E. (g/cm^2^)	*P*^c^	n	Adjusted BMD Mean ± S.E. (g/cm^2^)	*P*^c^	
eGFR (mL/min/1.73 m^2^)										
≥80^b^	885	**1.18** ± **0.14**	**0.001**	720	**1.20** ± **0.13**	**<0.0001**	156	1.10 ± 0.15	0.20	0.90
<80^b^	534	**1.21** ± **0.14**		381	**1.24** ± **0.12**		146	1.12 ± 0.14		
CCr (mL/min)										
≥78^b^	661	**1.22** ± **0.13**	**<0.0001**	545	**1.23** ± **0.12**	**<0.0001**	105	**1.14 ± 0.14**	**0.001**	0.24
<78^b^	758	**1.17 ± 0.14**		556	**1.20** ± **0.13**		197	**1.09** ± **0.14**		

Abbreviations: S.E., standard error; BMD, bone mineral density; eGFR, estimated glomerular filtrationrate; CCr, creatinine clearance rate.

Numbers in bold indicated statistical significant findings.

^a^Adjusted BMD was obtained from linear regression models adjusting for age, menopausal status (yes/no), body weight (for eGFR model only), hypertension, low-density lipoprotein cholesterol, fasting glucose, serum alkaline phosphatase, serum uric acid, height (for CCr model only), and use of Chinese herb (yes/no).

^b^The cutoff values of eGFR (80 mL/min/1.73 m^2^) and CCr (78 mL/min) were determined by maximizing the Youden’s index.

^c^*P*-values were obtained from Mann-Whitney U test.

**Table 4:** Association between renal function (eGFR or CCr) and BMD by the use of Chinese herb.

Renal function	Use of Chinese herb				*P*_*interaction*_
	**No (n = 1,296)**		**Yes (n = 120)**		
	n (L/H)	AOR (95% CI)	n (L/H)	AOR (95% CI)	
eGFR (mL/min/1.73 m^2^)					
≥80^a^	523/279	1.00	54/27	1.00	**0.03**
<80^a^	353/141	**0.62 (0.47**–**0.82)**	18/21	1.77 (0.72–4.36)	
CCr (mL/min)					
≥78^a^	451/153	1.00	37/18	1.00	0.57
<78^a^	425/267	**1.48 (1.14**–**1.94)**	35/30	1.18 (0.46–3.01)	

Abbreviations: L, low BMD; H, high BMD; BMD, bone mineral density; OR, odds ratio; CI, confidence interval; eGFR, estimated glomerular filtration rate; CCr, creatinine clearance rate.

All models were adjusted for age, body weight (for eGFR model only), menopausal status, hypertension, low-density lipoprotein cholesterol, fasting glucose, serum alkaline phosphatase, serum uric acid, and height (for CCr model only).

Numbers in bold indicated statistical significant findings.

^a^The cutoff values of eGFR (80 mL/min/1.73 m^2^) and CCr (78 mL/min) were determined by maximizing the Youden’s index.

**Table 5:** Adjusted mean BMD level by high and low renal function and the use of Chinese herb.

Renal function	Overall (n = 1,419)			Not use of Chinese herb (n = 1,296)			Use of Chinese herb (n = 120)			*P*_*interaction*_
	n	Adjusted BMD^a^ Mean ± S.E. (g/cm^2^)	*P*^c^	n	Adjusted BMD Mean ± S.E. (g/cm^2^)	*P*^c^	n	Adjusted BMD Mean ± S.E. (g/cm^2^)	*P*^c^	
eGFR (mL/min/1.73 m^2^)										
≥80^b^	885	**1.18** ± **0.14**	**0.001**	802	**1.19** ± **0.13**	**0.001**	81	1.17 ± 0.14	0.51	0.36
<80^b^	534	**1.21** ± **0.14**		494	**1.21** ± **0.14**		39	1.15 ± 0.16		
CCr (mL/min)										
≥78^b^	661	**1.22** ± **0.13**	**<0.0001**	604	**1.22** ± **0.13**	**<0.0001**	55	1.18 ± 0.14	0.28	0.57
<78^b^	758	**1.17** ± **0.14**		692	**1.17** ± **0.14**		65	1.15 ± 0.16		

Abbreviations: S.E., standard error; BMD, bone mineral density; eGFR, estimated glomerular filtrationrate; CCr, creatinine clearance rate. Numbers in bold indicated statistical significant findings.

^a^Adjusted BMD was obtained from linear regression models adjusting for age, menopausal status (yes/no), body weight (for eGFR model only), hypertension, low-density lipoprotein cholesterol, fasting glucose, serum alkaline phosphatase, serum uric acid, height (for CCr model only), and use of Chinese herb (yes/no).

^b^The cutoff values of eGFR (80 mL/min/1.73 m^2^) and CCr (78 mL/min) were determined by maximizing the Youden’s index.

^c^*P*-values were obtained from Mann-Whitney U test.
